# IMPLEMENTATION OF OTTAWA ANKLE RULES IN UNIVERSITY HOSPITAL EMERGENCY ROOM: PILOT STUDY

**DOI:** 10.1590/1413-785220233105e266034

**Published:** 2023-10-23

**Authors:** SACHA PUGLIESE SCHIPER, HUGO MAIA RODRIGUES, JOÃO EDUARDO LIMA ERNESTO REIS, MAYARA BRANCO E SILVA, MAURO DINATO, RODRIGO GONÇALVES PAGNANO

**Affiliations:** 1 Universidade Estadual de Campinas, Faculdade de Ciências Medicas, Departamento de Ortopedia, Reumatologia e Traumatologia, Campinas, SP, Brazil

**Keywords:** Ankle Sprain, Ankle Fractures, Ankle, Radiography, Traumatismos do Tornozelo, Fraturas do Tornozelo, Tornozelo, Radiografia

## Abstract

Ankle injuries are the most common musculoskeletal injuries in emergency rooms and are associated with a great social and economic impact. The need to request additional tests for ankle sprains is based on suspicion of fracture. The Ottawa Ankle Rules (OAR) establish criteria for ordering radiographs to avoid performing unnecessary examinations. Objective: To evaluate the implementation of the Ottawa Rules as a protocol for treating ankle sprains in the emergency department of a university hospital. Methods: This is a retrospective observational study, conducted over a period of three months before and three months after implementation of the protocol. Results: In the first phase, all patients complaining of ankle sprain underwent radiographs. In the second phase, after the application of the OAR, out of 85 patients evaluated, only 58 underwent complementary exams, showing a reduction of 31.8% in the request for imaging exams. There was no significant difference in fracture detection between the two groups (p=0.476). Conclusion: The OAR can be used as a tool in diagnosing ankle sprains, and their implementation reduced the request for imaging exams. *Level of Evidence III, Retrospective Comparative Study*.

## RESUMO

Os traumatismos de tornozelo são as lesões musculoesqueléticas mais comuns nas salas de emergência e estão associadas a um grande impacto social e econômico. A solicitação de exames complementares para a entorse de tornozelo baseia-se na suspeita de fratura. As Regras de Ottawa para Tornozelo (ROT) estabelecem critérios para a solicitação de radiografias com o intuito de evitar a realização de exames desnecessários. Objetivo: Avaliar a implementação das ROT como protocolo de atendimento das entorses de tornozelo no pronto-socorro de um hospital universitário. Métodos: Estudo observacional retrospectivo que visou comparar a solicitação de radiografias e a presença de fraturas três meses antes e três meses após a implantação do protocolo. Resultados: Na primeira fase, todos os pacientes com queixa de entorse de tornozelo realizaram radiografias. Na segunda fase, após aplicação das ROT, de 85 pacientes avaliados, apenas 58 realizaram exames complementares, apresentando uma redução de 31,8% na solicitação dos exames de imagem. Não houve diferença na detecção de fraturas entre os dois grupos (p=0,476). Conclusão: As ROT podem ser utilizadas como ferramenta no atendimento das entorses de tornozelo e sua implantação reduziu a solicitação de exames de imagem. *Nível de Evidência III, Estudo Comparativo Retrospectivo*.

## INTRODUCTION

Ankle injuries are the most common musculoskeletal injuries in emergency rooms and are associated with a major social and economic impact.^1^ ^)^ About 40% of all ankle injuries occur during sports.^2^ In soccer, basketball, and volleyball athletes, it accounts for about 10% to 15% of all injuries.^3^ In the United Kingdom, one in every 10,000 people have this condition and about 5,000 injuries occur per day.^4^ In the Netherlands, approximately 520,000 people suffer traumatic ankle injuries every year, of which 200,000 result from sports activity.^5^ Even among Brazilian amateur university athletes, a sprained ankle was the most common injury in non-contact exercises.^6^ ^)^ The management of ankle injuries is a daily routine in emergency departments and, although most patients undergo radiography, an ankle or midfoot fracture occurs in less than 15% of cases.^7^ It is estimated that US$500,000,000 is spent annually on ankle radiography in Canada and the United States.^8^ Around a third of the total costs spent on sports injuries are due to ankle sprains.^5^ ^)^ The diagnostic investigation of an ankle injury is the result of a semiological survey, a complete physical examination, and, when necessary, complementary resources.^3^ In the initial assessment, it is a priority to exclude serious complications, such as fractures that can mimic or even be associated with ligament injuries.^9^ In the 1980s, Stiell et al.^10^ conducted a pioneering study to develop clinical decision rules for requesting X-rays in acute ankle injuries. The work was conducted in the emergency department of two university hospitals in Canada and, in order to avoid unnecessary radiographs, the Ottawa Ankle Rules (OAR) were developed. The rules consider radiographic examination necessary only when there is pain in specific bone points or Inability to weight-bear at least four steps.^7^ ^), (^ ^10^ The OAR are active, validated, and accepted in numerous trauma care centers around the world. Studies have shown that the sensitivity in detecting fractures is approximately 100% for both malleolar and midfoot fractures.^7^ The negative predictive value is also 100%, meaning that the use of the protocol has proved useful in excluding the diagnosis of fractures.^11^

Therefore, this study aimed to evaluate the repercussions of the Ottawa Ankle Rules as a care protocol for ankle sprains in the emergency department, considering the number of ankle radiograph requests before and after their implementation, as well as their effectiveness in diagnosing ankle fractures.

## METHOD

This is a retrospective observational study that evaluated 98 medical records before protocol implementation over a 3-month period (07/01/2018 to 09/30/2018) and 85 medical records after implementation, also over a 3-month period (10/01/2018 to 12/31/2018). Approved by the Human Research Ethics Committee of our institution and registered in the Plataforma Brasil (CAAE 97588718.1.0000.5404). The evaluated patients met the inclusion criteria established for the study: age over 18, an acute traumatic event that had occurred less than 10 days ago, and no previous care or radiological examinations. Patients with chronic ankle pain after a sprain (more than three weeks), polytraumatized patients, patients with altered level of consciousness, and pregnant women were excluded from the evaluation.

The pre-implementation period was aimed at evaluating patients without the use of AOR. At this point, the patient answered the anamnesis, followed by the physical examination. After undergoing a semiologic evaluation, the examiner requested radiographs for the patients without pre-established standardized criteria. In a second moment, protocol was implemented The entire orthopedics team of the university hospital participated in a training class. The topics covered were applied anatomy, the main ankle injuries, the trauma mechanism of sprains, and the standardization of the Ottawa Rules for ankle sprains, with the aim of standardizing care for the entire emergency department personnel.

The post-implementation period was aimed at evaluating patients with a history of acute ankle sprain under the protocol guidelines. During the initial assessment, a clinical history was taken, a physical examination was conducted, and the need for radiographs was assessed according to the Ottawa Rules (Table 1).

**Table 1:** Ottawa Ankle Rules (AOR)

Request an ankle radiograph if pain in the malleolar region is associated with any of the following:	Request foot radiograph if pain in the midfoot is associated with any of the following:
A) Pain on bony tenderness over the posterior edge of lateral malleolus (6 cm)	A) Bony tenderness at the base of the fifth metatarsal
B) Pain on bony tenderness over the posterior edge of the medial malleolus (6 cm)	B) Bony tenderness at the navicular bone
C) Inability to weight-bear immediately and after clinical observation	C) Inability to weight-bear immediately and after clinical observation

Patients admitted to the emergency department complaining of an ankle sprain, both in the first and second phases of the study, experienced the same conditions of care and assessment, with all the propaedeutic resources offered by our service. The only difference was that the first group underwent a radiological study, with no defined protocol, whereas the second group only underwent complementary examinations after AOR indication.

The data was analyzed using the free software program R Core Team (2021), version 4.1.1 (2021-10-08). To test the hypothesis of independence between categorical variables, the Chi-square test or Fisher’s exact test was used. To test equal distributions for ordinal categorical variables, the Mann-Whitney test was used. The significance level adopted was 5% (p<0.05).

## RESULTS

In the cases of patients with ankle sprains evaluated, 98 records (53.5%) correspond to the pre-implementation phase, whereas 85 records (46.5%) refer to the post-implementation phase. The general characteristics of the studied groups, pre- and post-implementation, were compared and found to be statistically similar in terms of sex, age, and laterality (Table 2).

**Table 2:** Comparative analysis between the groups studied.

Characteristic	General n=183	PRE n=98	POST n=85	p-value
Sex				
Female	97 (53.01%)	52 (53.06%)	45 (52.94%)	1.000*
Male	86 (46.99%)	46 (46.94%)	40 (47.06%)	
Age group				
18-30	79 (43.17%)	42 (42.86%)	37 (43.53%)	0.651**
31-40	47 (25.68%)	22 (22.45%)	25 (29.41%)	
41-50	15 (8.2%)	11 (11.22%)	4 (4.71%)	
51-60	12 (6.56%)	6 (6.12%)	6 (7.06%)	
60+	30 (16.39%)	17 (17.35%)	13 (15.29%)	
Laterality				
RIGHT	100 (54.64%)	53 (54.08%)	47 (55.29%)	0.988*
LEFT	83 (45.36%)	45 (45.92%)	38 (44.71%)	

*Chi-square test; **Mann-Whitney test.

The first phase of the study found 52 females and 46 males, whereas the second phase found 45 females and 40 males. In both phases, the sex distribution was approximately 53% female patients and 47% male patients out of the total study population.

**Figure 1:**
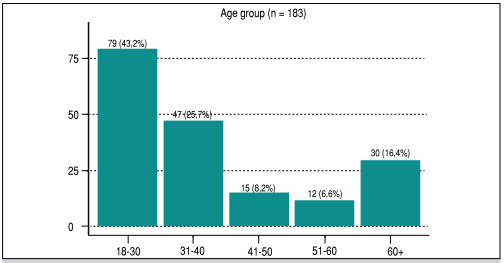
Distribution by age group (in years).

Furthermore, considering the total of 183 cases evaluated, 43.2% were in the 18-30 age group, 25.7% were 31-40 years old, and 6.5% were 51-60 years old. The highest frequency of ankle sprains was among adults aged 18-40, accounting for 68.9% of cases (Figure 1).

When assessing the laterality of the examinations of patients with sprains, 100 cases (54.6%) and 83 cases (45.4%) of injuries occurred in the right and left ankle, respectively.

**Figure 2:**
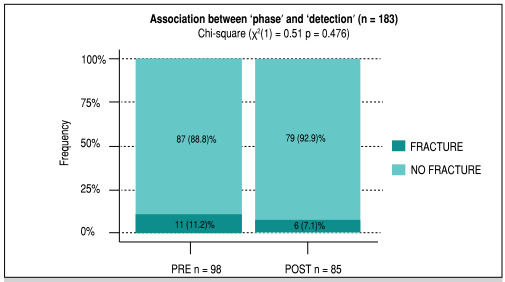
Association between phase and fracture detection.

Over the first three months of data collection, out of 98 patients assessed, 11 (11.2%) were diagnosed with a fracture. During the second phase of the study, out of 85 patients, 6 cases of fracture were diagnosed, which represents 7.1% of all the individuals assessed in the group, or 10.3% of the radiographed patients (Figure 2). The association was investigated using the Chi-square test of independence, which found no evidence of an association (p=0.476).

Regarding reassessments, in the first phase of the study, 15 patients (15%) returned to the service on their own initiative for reassessment due to persistent symptoms after the acute sprain episode. All 15 patients had already undergone radiographs on their first visit, and during the reassessment they underwent a further examination to confirm the diagnosis. However, in this second evaluation, it was observed that two patients (13%) out of 15 had a diagnosis of fracture, which went unnoticed in the first clinical examination, even though imaging tests had been conducted.

In the next phase, a total of five reassessments were conducted (5.8%). In contrast to the first phase, these patients were subjected to the protocol again and were only referred for further tests if they met the necessary AOR criteria. Out of these five patients, two were referred for radiography, but none had a change in their initial diagnosis. In addition, no fractures went unnoticed during the initial assessment.

In both reassessment phases, patients were not re-included in the sample.

**Figure 3:**
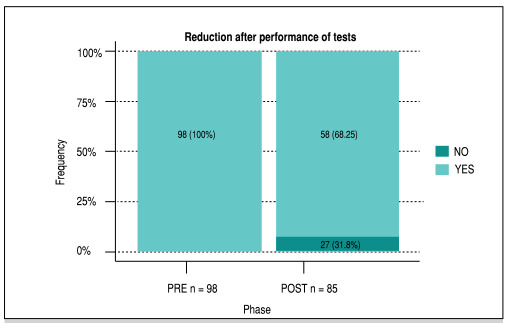
Examinations by phase.

At the end of the data collection, an analysis of the information regarding the request for imaging tests was generated. During the first phase, 98 patients were treated, and all (100%) underwent radiographs. During the second phase, after implementation of the AOR, out of the 85 patients evaluated, 58 (68.2%) were indicated for radiographs, which meant an absolute reduction of 31.8% in the number of requests for radiographs (Figure 3).

## DISCUSSION

The Ottawa Ankle Rules are objective criteria that allow to reduce the subjective component of clinical evaluation, providing specific and standardized indications for the performance of radiographs. The use of these criteria is simple, validated, and present high sensitivity and specificity.^9^

Heyworth^12^ states that these rules have transformed the way ankle sprain injuries are assessed. After appropriate training and adequate knowledge, they can be used by various healthcare providers.^12^ The reproducibility between examiners is high, which, added to its low cost and time of accomplishment, facilitates the management of ankle sprains. In our study, during the implementation phase, training of the entire orthopedic medical team was performed as recommended. The applicability among peers did not show difficulties, in line with the found data.

In 2003, Bachmann et al.^7^ confirmed that the Ottawa Ankle Rules accurately exclude ankle and midfoot fractures and can reduce the number of unnecessary radiographs by 30% to 40%. This showed compliance with our results, which presented a 31.8% reduction in the request for radiographs. Moreover, the patients who were reassessed did not present any change in the initial diagnosis, demonstrating the agreement and accuracy of the OAR protocol.

Before the development of OAR, Stiell et al.^10^ found that fewer than 15% of patients who presented to emergency departments with ankle sprains and received radiographs actually had fractures. The implementation of OAR reflects a decrease in hospital expenses since it reduces unnecessary tests, in addition to preventing exposure to ionizing radiation and optimizing the consultation time in the emergency room. Anis et al.^8^ highlighted that patients who did not undergo radiographs were discharged from the emergency room 36 minutes before the other patients.

A large sample study in the United States showed a reduction in spending of US$3 million per 100,000 patients annually after 90% of emergency units applied OAR.^13^ The potential savings from cutting overall hospital expenses are crucial, especially since public facilities struggle to meet the demands on their limited budgets.

During data collection, it was possible to observe a slight prevalence of females and young adults aged 18 to 30 years. Previous studies have shown a higher incidence of ankle sprains among females. The anatomical, hormonal, and neuromuscular differences between sexes do not necessarily explain the increase in this risk but should be considered for future studies. Moreover, lesions are more prevalent in the active population, especially among adolescents and young adults.^14^

## CONCLUSION

This study evidenced the feasibility of implementing the Ottawa Ankle Rules as a care protocol for acute ankle sprains. The data indicated a reduction in the number of radiography requests and the protocol was effective in excluding fractures, with high reproducibility between examiners. The reduction in hospital expenses, less exposure to radiation, and optimization of consultation time in the emergency room ensure that the implementation of the OAR protocol is an appropriate tool for the care of ankle sprains.
